# From bites to bytes: understanding how and why individual malaria risk varies using artificial intelligence and causal inference

**DOI:** 10.3389/fgene.2025.1599826

**Published:** 2025-05-16

**Authors:** Adèle Helena Ribeiro, Júlia M. P. Soler, Rodrigo M. Corder, Marcelo U. Ferreira, Dominik Heider

**Affiliations:** ^1^ Institute of Medical Informatics, University of Münster, Münster, Germany; ^2^ Institute of Mathematics and Statistics, University of São Paulo, São Paulo, Brazil; ^3^ Institute of Biomedical Sciences, University of São Paulo, São Paulo, Brazil; ^4^ Institute of Hygiene and Tropical Medicine and Global Health and Tropical Medicine Research Center, NOVA University of Lisbon, Lisbon, Portugal

**Keywords:** artificial intelligence, causality, causal modelling, malaria, infectious diseases, public health

## Abstract

With an estimated 263 million cases recorded worldwide in 2023, malaria remains a major global health challenge, particularly in tropical regions with limited healthcare access. Beyond its health impact, malaria disrupts education, economic development, and social equality. While traditional research has focused on biological factors underlying human-mosquito interactions, growing evidence highlights the complex interplay of environmental, behavioral, and socioeconomic factors, alongside mobility and both human and parasite genetics, in shaping transmission dynamics, recurrence patterns, and control effectiveness. This work shows how integrating Artificial Intelligence (AI), Machine Learning (ML), and Causal Inference can advance malaria research by identifying context-specific risk factors, uncovering causal mechanisms, and informing more effective, targeted interventions. Drawing on the Mâncio Lima cohort, a longitudinal, multimodal study of malaria risk in Brazil’s main urban hotspot, and related studies in the Amazon, we highlight how rigorous, data-driven approaches can address the substantial variability in malaria risk across individuals and communities. AI-driven methods facilitate the integration of diverse high-dimensional datasets to uncover intricate patterns and improve individual risk stratification. Federated learning enables collaborative analysis across regions while preserving data privacy. Meanwhile, causal discovery and effect identification tools further strengthen these approaches by distinguishing genuine causal relationships from spurious associations. Together, these approaches offer a principled, scalable, and privacy-preserving framework that enables researchers to move beyond predictive modeling toward actionable causal insights. This shift supports precision public health strategies tailored to vulnerable populations, fostering more equitable and sustainable malaria control and contributing to the reduction of the global malaria burden.

## Introduction

Malaria remains a major health challenge, particularly in tropical and subtropical regions facing poverty, limited healthcare access, and harsh environments, such as the Amazon rainforest. In 2023, an estimated 263 million malaria cases occurred across 83 countries and territories – 37 million more than in 2015 (World Health Organization, 2024). Conflicts, humanitarian crises, climate change, drug and insecticide resistance, and resource constraints are among the threats to malaria control efforts.

*P. falciparum* predominates in sub-Saharan Africa, causing the most severe form of human malaria (Poespoprodjo et al., 2023). *P. vivax* is the most geographically widespread parasite, responsible for over 80% of infections in the Amazon and causing recurrent infections. Malaria’s impact extends beyond health, disrupting education, hindering economic growth, straining healthcare systems, and perpetuating poverty. Effective control is crucial for public health, equity and global prosperity, requiring a shift from the traditional human-mosquito transmission model to a broader understanding of biological, environmental, and socioeconomic factors.

We take as an example the Mâncio Lima cohort study, which focuses on urban malaria in the Brazilian Amazon (Johansen et al., 2021). Approximately 20% of households in Mâncio Lima, Brazil’s primary urban hotspot near the Peruvian border, were randomly selected from census data, resulting in 2,774 participants tested for malaria parasites during seven cross-sectional surveys (2018–2021) using conventional microscopy and highly sensitive, species-specific molecular techniques (Rodrigues et al., 2024). The study gathered data on demographics, health, housing conditions, occupation, lifestyle, and mobility, alongside blood samples for human genetics research, including genome-wide association studies.

Complementary longitudinal studies across Latin America have investigated the genomic diversity of *P. vivax* and *P. falciparum* (de Oliveira et al., 2020; Cabrera-Sosa et al., 2024; Kattenberg et al., 2024). Conducted in both urban and rural areas around Mâncio Lima (2018–2021) and the Peruvian Amazon (2007–2020), these studies support integrative genomic surveillance to track transmission intensity, imported cases, and drug resistance markers. By linking human and parasite data across diverse settings, these efforts support research on malaria dynamics and the evolution of key traits, such as virulence, resistance, and local adaptation, while accounting for ecological and socio-demographic variation.

The Mâncio Lima cohort has yielded several insights (Corder et al., 2019; Corder et al., 2020b; de Oliveira et al., 2020; Corder et al., 2023; Rodrigues et al., 2024). Of 11,730 samples screened using molecular methods, 4.0% were positive for *P. vivax* and 0.9% for *P. falciparum*, whereas standard microscopy detected much lower rates (0.4% for *P. vivax* and 0.2% for *P. falciparum*) (Rodrigues et al., 2024). Despite the low prevalence, *P. vivax* infections were recurrent (Corder et al., 2020a; Corder et al., 2023), with model simulations indicating that 20% of individuals at highest risk of infection accounted for 86% of the infection burden (Corder et al., 2020b). This highlights that malaria burden is often heterogeneously distributed within communities, following the 20/80 rule, where approximately 20% of individuals carry 80% of infections (Corder et al., 2023). Adult men face the highest risk (Corder et al., 2019), and most laboratory-confirmed infections were asymptomatic (Rodrigues et al., 2024). Human mobility between urban and rural areas appears to sustain malaria transmission (Johansen et al., 2020). Additionally, genetic analyses of *P. vivax* revealed diverse, spatially and temporally structured lineages, highlighting heterogeneous transmission dynamics across different settings (de Oliveira et al., 2020; Kattenberg et al., 2024). In contrast, *P. falciparum* exhibited lower genetic diversity and stronger temporal clustering, indicating localized and time-limited transmission (Cabrera-Sosa et al., 2024).

Despite these heterogeneities, malaria prevalence in Mancio Lima declined significantly from 2018 to 2021, likely due to extensive control and treatment efforts, including widespread indoor residual spraying, distribution of insecticide-treated bed nets, active case testing, and free treatment programs. Sustaining and advancing this progress requires improved identification of high-risk groups for optimizing resource distribution and implementing tailored interventions. A key challenge is understanding why some individuals repeatedly contract *P. vivax* while others remain uninfected. Clinically, such recurrences can lead to severe complications, including anemia, particularly among vulnerable groups, such as children and pregnant women (Pincelli et al., 2021). Economically, this heterogeneity complicates policy design. The 20/80 rule suggests that targeting high-risk individuals could maximize impact (Corder et al., 2023). Additionally, malaria has emerged as a zoonotic threat. *P. simium*, a parasite of non-human primates, has caused infections in humans in southeastern Brazil, where *P. vivax* is rare (de Oliveira et al., 2021a; b). Distinguishing between human and zoonotic parasites is critical for evaluating interventions and preparing for future outbreaks.

To elucidate the multifaceted dynamics underlying malaria risk, we propose a synergistic integration of AI, ML, and causal inference. This combination enables not only the identification of high-risk groups but also the discovery of causal mechanisms driving individual variability in malaria susceptibility. By leveraging cutting-edge methods, we can move beyond predictive modeling toward causal understanding, thereby informing the development of optimized, targeted interventions. Our approach relies on the integration of high-dimensional, multimodal datasets such as those from the Mancio Lima cohort and other regional studies – including data on malaria episodes, clinical, behavioral, socioeconomic, environmental, and genetic factors. This rich data landscape enables the identification of structured patterns and interpretable representations that explain malaria risk and transmission dynamics. Causal inference methods that account for latent confounding and selection bias are essential to distinguish causal drivers from spurious associations, enabling robust estimation of intervention effects under real-world conditions. Ultimately, this framework will support precision public health by ensuring that prevention, control, and treatment strategies are both timely and tailored to those most at risk, maximizing impact and equity (Khoury et al., 2015).

## Bridging AI and causality for targeted malaria interventions

AI and ML have driven significant advancements in medicine and public health (MacEachern and Forkert, 2021) due to their ability to model complex relationships and uncover subtle patterns in high-dimensional, heterogeneous datasets. These methods have been successfully applied across various medical domains (Theodosiou and Read, 2023), including infectious disease research, such as AMR prediction (Ren et al., 2022), zoonotic disease detection (Ren et al., 2024), and biomarker discovery in malaria (Jung et al., 2023).

**FIGURE 1:**
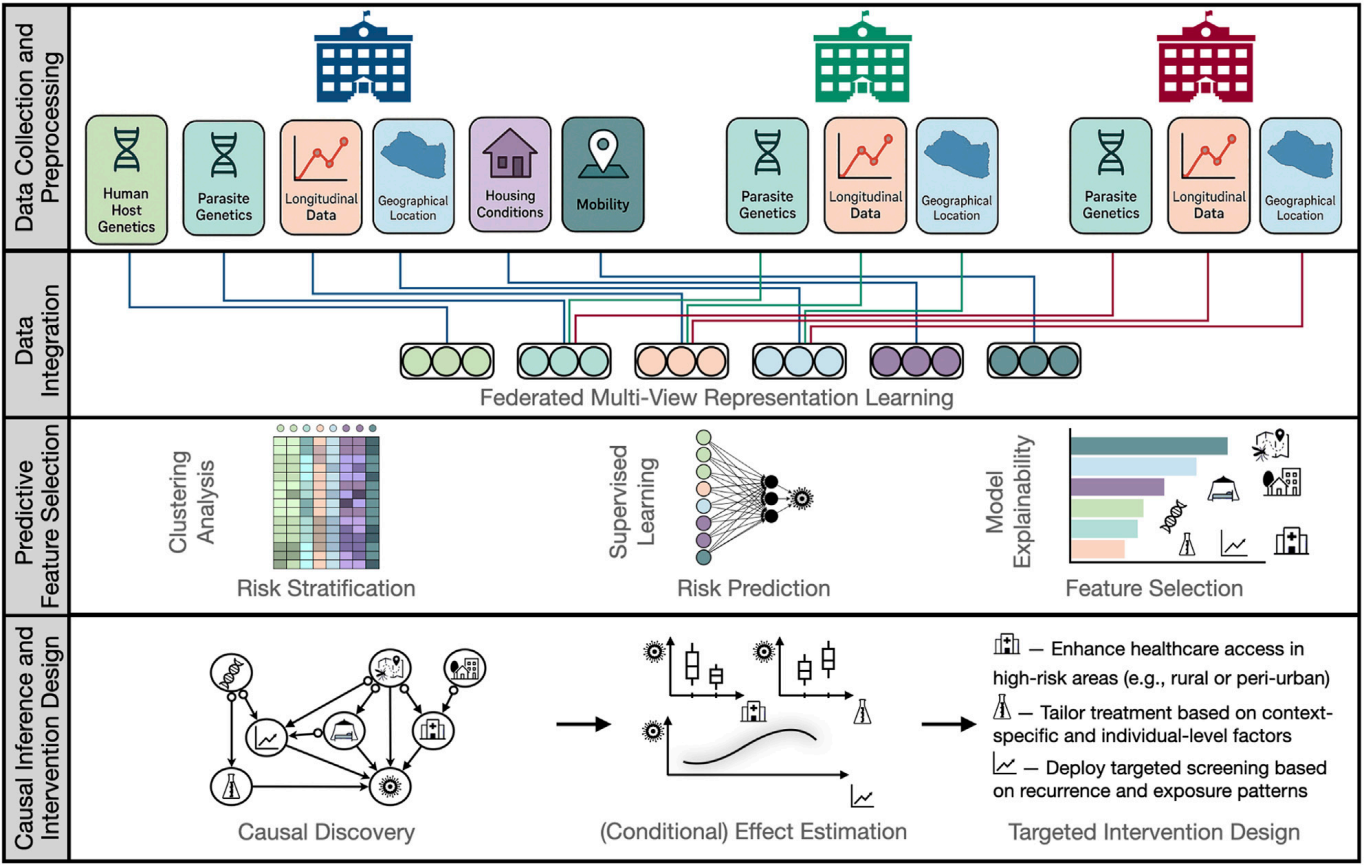
AI and Causal Inference Pipeline for Targeted Intervention Design in Malaria Research. The pipeline begins with Data Collection and Preprocessing, including anonymization, harmonization, and normalization of multimodal, multi-center data. In Data Integration, federated multi-view representation learning generates low-dimensional embeddings that capture both within- and cross-modal patterns while maintaining data privacy. Predictive Feature Selection uncovers latent risk profiles and selects interpretable features that predict malaria risk both globally and within specific subgroups. Finally, Causal Inference and Intervention Design applies causal discovery to reveal mechanisms underlying the selected features – e.g., treatment regimens, prior infection history, genetic predispositions, bed net usage, healthcare access, urban vs. rural residence, and proximity to mosquito breeding sites. Causal effect estimation tools then quantify the (conditional) impact of specific interventions (e.g., increasing healthcare access, personalizing treatments, or implementing targeted screening) from observational data, supporting precision public health strategies for effective malaria prevention, treatment, and control.

In malaria research, AI and ML provide powerful tools to disentangle complex, often hidden dependency structures and enable precise individual risk stratification. The pipeline (Figure 1) begins with data collection and preprocessing, crucial for multimodal, heterogeneous, and sensitive data such as genomic and socio-behavioral information. Ensuring data privacy and quality through anonymization, harmonization, imputation, and normalization – while following FAIR principles (Findable, Accessible, Interoperable, Reusable) (Kush et al., 2020) – is essential for robust model development. In multi-center studies, federated learning supports privacy-preserving collaboration by enabling joint analysis without exchanging raw data (McMahan et al., 2017; Tajabadi et al., 2023; Tajabadi et al., 2024).

Multi-view representation learning approaches, such as multimodal variational autoencoders, enable data integration by generating low-dimensional latent embeddings that retain modality-specific features while capturing cross-modal dependencies (Guo et al., 2019). Clustering these embeddings can reveal subgroups of individuals with shared but not directly observed risk profiles, shaped by common exposures or susceptibilities (Jaeger et al., 2023). This step can be enriched through co-clustering, which jointly identifies groups of individuals and co-varying variables, highlighting context-specific drivers of malaria vulnerability (Govaert and Nadif, 2013). Moreover, federated representation learning and clustering (Zhang et al., 2023; Pedrycz, 2021) support robust and generalizable predictions across distributed, heterogeneous datasets. To enhance interpretability and inform downstream modeling, cluster-aware feature selection (Wang and Allen, 2021) identifies both globally predictive variables and those particularly informative within specific subgroups. These selected features and representations are then used to predict individual malaria risk, forming a cohesive and interpretable AI-driven framework for risk assessment.

While essential, high predictive accuracy alone is not sufficient to uncover the underlying data-generating mechanisms or support meaningful, actionable interventions. This is particularly true in biomedical and epidemiological research, where data are largely observational and vulnerable to multiple sources of bias. In malaria research, for example, unmeasured factors such as socio-economic status, mobility patterns, or environmental exposures can confound associations between risk factors and outcomes. Selection bias is also widespread due to underreporting, especially in remote regions or among asymptomatic individuals. If not properly addressed, these biases can reinforce existing health disparities and lead to interventions that are ineffective or even harmful.

Causal inference provides a principled framework to uncover cause-and-effect relationships and mitigate the impact of bias in observational studies (Pearl, 2009). It enables the estimation of the effect of interventions with a level of rigor comparable to randomized controlled trials. Several approaches exist, including the Potential Outcomes Framework (Rubin, 1974), Causal Machine Learning (van der Laan and Rubin, 2006; Feuerriegel et al., 2024), and Instrumental Variables (Angrist et al., 1996), also known in genetics as Mendelian Randomization (Haycock et al., 2016; Ribeiro et al., 2016). However, these frameworks rely on strong, sometimes unverifiable assumptions – such as the absence of latent confounding or availability of valid instruments – which are often violated in real-world settings.

In response, data-driven causal discovery methods within Pearl’s framework have emerged as robust alternatives. Algorithms such as Fast Causal Inference (FCI) (Zhang, 2008) and its variants can recover causal structures directly from observational data, even in the presence of unmeasured confounding and selection bias. Notably, AnchorFCI (Ribeiro et al., 2024) enhances robustness and discovery power by strategically selecting and integrating reliable anchor variables – such as genetic variants – that are known not to be influenced by the variables of interest (e.g., clinical or sociodemographic factors). These methods infer a Partial Ancestral Graph (PAG) representing causal relationships shared across all models supported by the data, thus revealing the true data-generating processes. This enables the identification of key factors – e.g., use of insecticide-treated bed nets, housing conditions, or access to healthcare – that causally influence malaria risk and can be targeted by interventions. By applying causal effect identification algorithms to the resulting PAG, we can then quantify the isolated or combined impact of specific interventions, based solely on observational data (Perković et al., 2018; Jaber et al., 2022). This fully data-driven causal pipeline supports the development of more robust, transparent, and socially responsible interventions, providing a clearer pathway for addressing malaria risk in diverse settings.

A key strength of constraint-based causal discovery approaches such as FCI and its variants lies in their flexibility to account for mixed-type variables and complex dependency structures by adapting conditional independence tests. This is particularly important for analyzing malaria datasets, which typically comprise a mix of continuous, ordinal, categorical, and count variables, along with non-independent observations arising from genetic relatedness, repeated measures, household clustering, and spatial correlations. Conditional independence tests that account for such complexities can be constructed using generalized mixed models, which incorporate structured covariance and random effects to model known or inferred dependencies (Ribeiro and Soler, 2020). These tests can also be extended to federated learning settings, enabling collaborative, privacy-preserving causal discovery. Furthermore, causal discovery at the level of variable clusters – either predefined or learned through representation learning and clustering – can yield more interpretable insights into the interactions among biological, behavioral, and environmental risk factors for malaria (Anand et al., 2023).

## Discussion

Progress toward malaria elimination in regions such as the Amazon requires a deep understanding of the intricate factors driving infection risk and recurrence. The Mâncio Lima cohort and regional studies offer a unique opportunity to uncover malaria dynamics by combining comprehensive data on human hosts, parasites, and their environments. However, the inherent complexity and heterogeneity of these datasets demand analytical frameworks that extend beyond traditional epidemiological or statistical approaches.

By integrating AI, ML, and causal inference, we move toward a more holistic strategy that not only accurately identifies high-risk individuals but also elucidates the causal mechanisms underlying malaria transmission and infection. This shift from descriptive and predictive modeling to causal reasoning enables the development of optimized, targeted interventions and lays the foundation for precision public health strategies that are not only more effective but also more equitable. Federated learning further supports this approach by enabling collaborative analysis across diverse regions without compromising data privacy. Together, these methodologies empower local health systems to respond more precisely and efficiently and contribute meaningfully to global control efforts.
